# Supplementary material for: A behaviourally informed chatbot increases vaccination rates in Argentina more than a one-way reminder
Source: Nat Hum Behav. 2024 Oct 18;8(12):2314–21. doi: 10.1038/s41562-024-01985-7 (PMC11659163; doi:10.1038/s41562-024-01985-7)
Supplement: Supplementary file 1 — Supplementary Fig. 1, Tables 1–6 and Text. [file 41562_2024_1985_MOESM1_ESM.pdf]

nature human behaviour

Supplementary information https://doi.org/10.1038/s41562-024-01985-7

A behaviourally informed chatbot increases vaccination rates in Argentina more than a one-way reminder

In the format provided by the authors and unedited

Table of contents

1. Supplementary Text **2**
1.1. Exploratory research 2
1.2. Access to the chatbot 2
1.3. Implementation of a rolling trial design to account for WhatsApp's message limits 3
1.4. Building trust in the chatbot 4
1.5. One-way message content 5
1.6. Vaccination eligibility criteria 5
1.7. Masking 6
1.8. Balance tests 7
1.9. Robustness checks 7
1.10. Subgroup analysis 8
1.11. Estimated costs of the intervention 8
2. Supplementary Figures **9**
3. Supplementary Tables **10**

1. Supplementary Text

1.1. Exploratory research

Our exploratory qualitative research activities included in-depth interviews with 8 healthcare workers and 12 patients receiving COVID-19 vaccines at health centres; 1 focus group with the general population; 2 focus groups with healthcare workers in Chaco; and a desk review of more than 200 Google reviews of health centres in the province.

We had two primary aims for the qualitative research: (1) identify current perceived barriers to accessing COVID-19 vaccination within the local context, and (2) understand the reactions, attitudes, and concerns of participants regarding the chatbot and its potential use in Chaco. Additionally, the interviews with healthcare personnel aimed to deepen our understanding of current procedures and the functioning of the vaccination service in the province.

Regarding the first aim, two common themes emerged across participants: (1) Anti-vaccine attitudes and vaccine hesitancy were not the primary factors contributing to the low rates of booster dose uptake. A significant portion of the population had received their first and second doses, and there were no widespread reports of adverse side effects or substantial activism against the vaccine in the local context; (2) Despite public media campaigns, many individuals were unaware of when and where to receive their booster doses. This lack of awareness was attributed to the absence of personalised information about eligibility for subsequent doses and the changing locations of vaccination centres over time as the vaccine rollout evolved. These learnings formed the basis of the functionalities that we built into the chatbot. Information gathered from healthcare providers confirmed that attendance at vaccination centres was spontaneous, with no prior appointments made by individuals.

Concerning reactions to the chatbot, both the general population and healthcare participants expressed positive impressions about the concept and functionalities of the chatbot. The primary concern regarding the chatbot was related to its security, given previous instances of fraudulent activities on the WhatsApp platform. In response, we aimed to ensure the chatbot was perceived as reliable and trustworthy (see below for further details).

1.2. Access to the chatbot

We coded the messaging platform to ensure that only those assigned to the behaviourally-informed chatbot group could access the chatbot. If any individual in the control group sent a WhatsApp message to the Ministry of Health's phone line, they received a message which stated '*Buenos días* 👋 *Este es un chatbot del Ministerio de Salud de Chaco. Actualmente el chat no está disponible para todos, mientras lo probamos. Esperamos hacer este servicio disponible a más Chaqueños en el futuro'* ['Good morning 👋 This is a chatbot from the Chaco Ministry of Health. Currently the chat is not available to everyone, while we test it.

We hope to make this service available to more Chaqueños in the future']. This was also the message sent to any individual from outside the set of trial participants who contacted the Ministry of Health's phone line. If any individual in the one-way message group sent a WhatsApp message to the Ministry of Health's phone line they received a message saying '*Gracias por comunicarte con nuestro servicio, no tenemos más opciones para mostrarte'* ['Thank you for contacting our service, we have no other options to show you'].

1.3. Implementation of a rolling trial design to account for WhatsApp's message limits

WhatsApp imposes limits on the number of 'business-initiated' messages that can be sent from a WhatsApp Business Account on any given day. Business-initiated messages refer to any message sent from the WhatsApp Business Account after a 24-hour period where the user has not interacted with the phone line. We had two types of business-initiated messages: 1) the first message sent to a trial participant in either the chatbot group or the one-way message reminder group, 2) the reminder message sent one day before the chosen date of vaccination for individuals in the chatbot group who completed the chatbot flow. Outbound messages from our phone line which responded to the user's inputs through the chatbot flow do not count as business-initiated messages.

At the time, WhatsApp operated a tiered system, where any new Business Account begins in Tier 1 and can graduate up to Tier 3. Accounts that are in Tier 1, Tier 2 and Tier 3 can send up to 1,000, 10,000 and 100,000 business-initiated messages per day respectively. Graduation through the tiers is dependent on your quality rating (which among other things depends on the number of phone numbers that block your phone line), and whether you are using your existing limit. Our WhatsApp Business Account graduated from Tier 1 to Tier 2 on day 4 of the trial, and from Tier 2 to Tier 3 on day 7 of the trial. This progression through the tiers allowed us to enrol all participants in the trial over a 2 week period from September 8th to September 21st 2022.

Given this restriction, we had to implement the trial on a rolling basis. In addition, we had to ensure that: 1) all reminder messages to chatbot group participants the day before their chosen vaccination date (i.e., those who had completed the chatbot flow and had a reminder automatically programmed) were sent before we reached the message limit, and 2) the number of participants who entered the trial on any given day was balanced across trial arms.

To do this, we coded the platform to use the following implementation procedure on each day of the trial:

- First, reminder messages were sent to any behaviourally-informed chatbot group participant who had previously been enrolled in the trial, completed the chatbot flow and selected the following day as the date they would like to get vaccinated. This ensured that no chatbot group participant who had planned their vaccination would miss out on their reminder due to WhatsApp's message limits.

- Second, the number of remaining messages within the business-initiated limit (based on the current Tier of our WhatsApp Business Account) was calculated.
- Next, this number was divided by two, to give the number of new trial participants in each of the chatbot and one-way message reminder groups who would be enrolled into the trial that day.
- Before the start of the trial, individuals in each trial arm had been randomly sorted into a list. Each day, the next set of participants to be enrolled into the chatbot or one-way message reminder groups were taken from the top of that list, starting from where enrolment had finished the previous day.
- If any participant in any trial arm sent a WhatsApp message to the Ministry of Health's phone line before they had been enrolled into the trial, they received the default message stating "Good morning 👋 This is a chatbot from the Chaco Ministry of Health. Currently the chat is not available to everyone, while we test it. We hope to make this service available to more Chaqueños in the future". They later received their assigned message on the day that they were enrolled into the trial.
- If a business-initiated message failed to send to any trial participant on a given day, they were moved back to the top of the list so that they could be tried again the following day.

Individuals in the chatbot and one-way message reminder groups were therefore enrolled into the trial on the day that they were sent the first message of the behaviourally-informed chatbot flow or the one-way message respectively. If the first message failed to send to an individual, they were enrolled into the trial on the date on which the first message successfully sent, or, if the message never successfully sent, the last unsuccessful attempt.

We needed to ensure that we had the same number of control group participants entering the trial on any given day as the chatbot and one-way message reminder groups to make sure that the 4-week outcome observation window was consistent across all arms throughout the trial. To do this, since the control group does not receive any messages, we enrolled participants into the trial each day using the randomly generated pre-assigned order mentioned above.

More specifically, we randomly assigned a number to each individual in the control group during randomisation before the trial launch. At the conclusion of the trial, we assigned individuals from the control group to the 'control group for each day' to match the number of chatbot and one-way message reminder participants enrolled into the trial each day. For example, if there were 500 individuals in each of the chatbot and one-way message reminder groups on Day 1 and 400 in each of the chatbot and one-way message reminder groups on Day 2, we assigned participants in the control group who were randomly assigned the numbers 1-500 to become the 'Day 1 control group', individuals numbered 501-900 to become the 'Day 2 control group' and so on.

<u>1.4. Building trust in the chatbot</u>

We aimed to build trust in the chatbot in several ways. First, all messages were sent from the official Ministry of Health phone line, with the Ministry of Health logo appearing in the top left corner of the user's screen. Second, this phone line was verified by WhatsApp, meaning that a verified WhatsApp green tick appeared in the top right of the user's screen. Third, to reduce the chance that users believe the messages were spam we explicitly informed them in the first chatbot message that they would not be contacted again if they did not reply to the message, and asked for their consent to receive further messages. Finally, we pinned a post at the top of the Ministry of Health's facebook page stating (in Spanish): "We inform you that the Ministry of Health of the Province of Chaco is conducting a pilot test of a new chatbot service with messaging via WhatsApp to provide information to the public about COVID-19 vaccination. We confirm that the messages sent from the number +54 362 4 600964 come from the Ministry of Public Health and are legitimate."

1.5. One-way message content

Individuals in the one-way message group received a single WhatsApp message encouraging them to get the next dose of the COVID-19 vaccination. The message was sent from the same phone line (the official Ministry of Health phone line) as the chatbot, which had the Ministry of Health logo and WhatsApp verified green tick.

The message read: '*Te recordamos que es importante completar el esquema de vacunaciones contra Covid-19 y recibir todas las dosis de refuerzo. Con el paso del tiempo, tus defensas contra el virus disminuyen. Refuerza tu vacuna y no perdás tu protección contra el virus! En la página web oficial del Ministerio de Salud podés encontrar más información sobre cómo vacunarte*' ['We remind you that it is important to complete the Covid-19 vaccination schedule and receive all booster doses. Over time, your defences against the virus decrease. Strengthen your vaccine and do not lose your protection against the virus! On the official Ministry of Health website, you can find more information on how to get vaccinated']. The motivational message, which aimed to invoke loss aversion, was also included in the first message of the chatbot.

Supplementary Table 2 outlines the details of the chatbot.

1.6. Vaccination eligibility criteria

We only included individuals who were eligible to receive their next dose of the COVID-19 vaccine in our trial sample. To do this, we used the most up-to-date vaccine eligibility criteria provided to us by the Ministry of Health. Vaccine eligibility was based on the initial vaccination an individual received, the time period they must wait to receive the next dose, and personal characteristics such as age and immunosuppressed status, as outlined below. At this point in the pandemic, vaccinations from different manufacturers were used interchangeably for second and booster doses.

Moderna, Pfizer or Sputnik: If an individual's first dose was the Moderna, Pfizer or Sputnik vaccine, they were eligible for their second dose after 21 days. An individual had completed their full course once they had received their first two doses of these vaccines. After completing their full course, individuals were eligible to receive their first booster dose after 120 days. After receiving their first booster, individuals were eligible to receive their second booster dose after 120 days.

AstraZeneca: If an individual's first dose was the AstraZeneca vaccine, they were eligible for their second dose after 8 weeks. An individual had completed their full course once they had received their first two doses of these vaccines. After completing their full course, individuals were eligible to receive their first booster dose after 120 days. After receiving their first booster, individuals were eligible to receive their second booster dose after 120 days.

Sinopharm: If an individual's first dose was the Sinopharm vaccine, they were eligible for their second dose after 21 days. In most cases, 2 doses of Sinopharm was the full course (meaning an individual was eligible for their first booster dose). However, there was an exception for people over the age of 50 and/or considered immunosuppressed. These individuals were eligible for their third doses after 28 days, and only after receiving this dose had completed their full course. After completing their full course, individuals were eligible to receive their first booster dose after 120 days. After receiving their first booster, individuals were eligible to receive their second booster dose after 120 days. For people receiving a 3-dose full course Sinopharm vaccine, the first and second booster corresponded to their fourth and fifth doses respectively.

Cansino: If an individual's first dose was the Cansino vaccine, they were considered to have completed their full course of the vaccine. This vaccine was primarily administered to vulnerable populations, for example, people experiencing homelessness. After completing their full course, individuals were eligible to receive their first booster dose after 120 days. After receiving their first booster, individuals were eligible to receive their second booster dose after 120 days.

1.7. Masking

We do not expect participants to be aware that they are in a trial where different individuals were exposed to different conditions, meaning participants were blind to the intervention. The primary outcome measure used data from an administrative database and so did not require new data collection. However, the study team was not blind to group assignments. Whilst the Ministry of Health already had consent to contact individuals in the databases used to create the eligible sample, we also asked participants in the chatbot trial arm if they consented to continue with the chatbot in the first message.

1.8. Balance tests

In Supplementary Table 3, we tested whether the three trial arms were balanced in terms of the: sex and age of participants, the number of COVID-19 vaccine doses they had received before the launch of the trial, the days since their previous dose of the COVID-19 vaccine and how long after the launch of the trial they received the first message. In each case, we conducted a pairwise comparison between each of the three trial arms, meaning a total of fifteen balance tests. Details of the tests used in each case are given in column (1). There were no statistically significant imbalances, and the differences between trial arms were very small in real terms in all cases.

In Figure 3, which plots the number of vaccinations per day throughout the rollout of the COVID-19 vaccine starting from February 2021 for each of the trial arms, we similarly see an almost identical trend between the chatbot, one-way message reminder and control groups right up until the launch of the trial in September 2022.

1.9. Robustness checks

In Supplementary Table 5, we conducted a series of robustness checks on the primary regression analysis. These robustness checks were not part of the pre-specified analysis plan. In columns (1) to (4) we made changes to the regression specification: dropping all control variables except the key demographic controls (sex and age), dropping all control variables, estimating using a linear probability model, and estimating using a Probit model respectively. In all cases, the coefficients on both the one-way message reminder and chatbot treatment variables barely changed in size or statistical significance, and the difference in the effect of the chatbot from the one-way message reminder was statistically significant (p-value < 0.001). To correct for rare events in binary data, in columns (5) and (6) we use King and Zeng's correction for logistic regression and Firth's penalised likelihood approach, respectively. Again, the results are almost identical.

In columns (7) and (8), we changed the observation window for the outcome measure. In column (7), our dependent variable was a binary variable which takes the value 1 if the individual got their next dose of the COVID-19 vaccine within 2 weeks, and in column (8) we used a 6 week observation window. This logistic regression was otherwise identical to the primary regression analysis. As expected, we found slightly smaller percentage point increases in vaccination from the one-way message reminder and chatbot in the shorter observation window, and slightly larger percentage point increases of both in the longer observation window, compared to the 4 week observation window used in our main results. In all cases, the effect of the one-way message reminder and the chatbot was statistically significant, as was the difference between them (p-value < 0.001).

1.10. Subgroup analysis

In Supplementary Table 6, we conducted subgroup analysis by the number of existing doses the individual had received before the start of the trial. This subgroup analysis was part of the pre-specified analysis plan. The behaviourally-informed chatbot and one-way message both increased vaccination for all three groups (1 dose, 2 doses, 3 doses). Whilst the effect of the chatbot was larger than the one-way message in all cases, this difference was only statistically significant for individuals who had previously received 2 or 3 doses of the vaccine (p-values = 0.671, <0.001, <0.001 respectively). However, there were only 30,208 individuals with one prior dose of the vaccine (12% of the sample). Only 1,616 individuals had received 4 doses of the vaccine before the launch of the trial, and so we did not run a separate regression for that subgroup.

In Supplementary Table 4, we conducted subgroup analysis by two key demographic characteristics: sex and age. This subgroup analysis was not part of the pre-specified analysis plan. The behaviourally-informed chatbot and one-way message reminder increased vaccination for both men and women (with similar effect sizes), and the chatbot performed significantly better than the one-way message in both cases (p-value <0.001); see columns (1) and (2). Similarly, the chatbot and one-way message reminder increased vaccination for all age-groups (18-29, 30-49, 50+), and the chatbot performed significantly better than the one-way message for all age groups (p-values 0.001, <0.001, <0.001 respectively); see columns (3)-(5).

Whilst the effect size in percentage points was larger for older age groups, we caution that this could reflect differential selection into the trial sample in different age groups (e.g., a different subgroup of the population in older age-groups by some unobservable characteristic had access to WhatsApp) rather than a true difference in the effect of the chatbot at different ages.

1.11. Estimated costs of the intervention

We estimate that the intervention costs 46 USD per additional vaccination. We arrive at this figure by first subtracting research costs from the total project costs. We divide the remaining implementation costs (177,000 USD, which includes staff salaries) by the number of additional vaccinations the chatbot would have caused had it been distributed to the full sample rather than withholding the chatbot from two trial arms for research purposes. Given that the chatbot caused an additional 1,300 vaccinations compared to the control group, we expect that it would have caused an additional 3,900 vaccinations had it been distributed to the full sample.

2. Supplementary Figures

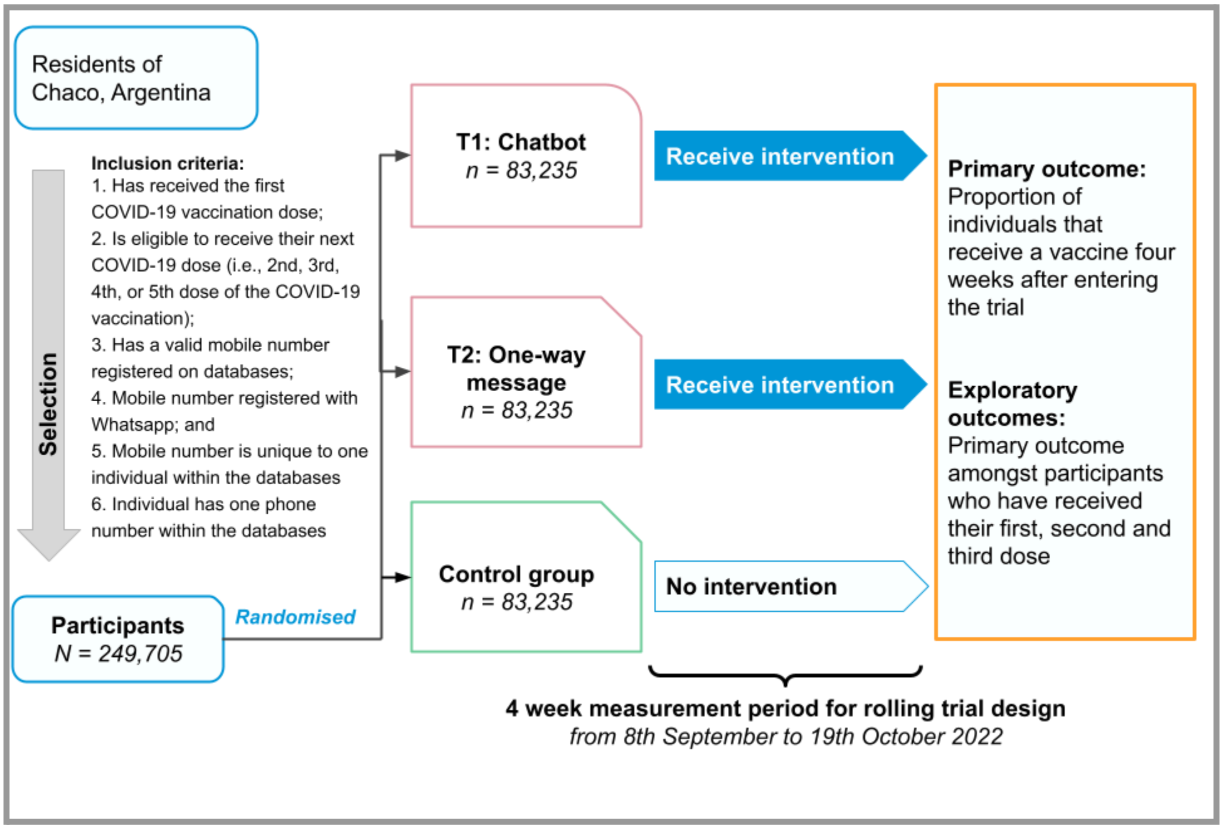

Supplementary Figure 1. Study recruitment, randomisation, implementation and measurement overview.

3. Supplementary Tables

	Country	URL	Developer / Provider
1	Albania	https://www.unicef.org/kosovoprogramme/press-releases/unicef-and-who-launch-healthbuddy-app-online-interactive-digital-tool-provides	UNICEF and WHO (HealthBuddy+)
2	Argentina	http://buenosaires.gob.ar/boti	Regional government
3	Armenia	https://itel.am/en/news/11986	News platform
4	Australia	https://www.headtohealth.gov.au/about/news-and-announcements/have-you-tried-sam-the-chatbot	National government
5	Austria	https://digitales.wien.gv.at/en/ai-against-covid-19-disinformation/	Regional government
6	Azerbaijan	https://www.undp.org/azerbaijan/press-releases/whatsapp-bot-launched-government-and-undp-keep-azerbaijans-citizens-date-latest-covid-19-info	UNDP
7	Bahrain	https://healthalert.gov.bh/uploads/xhybtpyu_zea.pdf	National government
8	Bangladesh	https://archive.dhakatribune.com/technology/2020/03/26/new-chatbot-to-	Private organisation(s) or individual(s)
9	Bolivia	https://saluddigital.com/en/plataformas-digitales/servicios-de-salud-en-la-paz-bolivia-presentaron-una-chatbot-de-consultas-y-diagnosticos-en-linea-para-personas-con-sintomas-de-covid-19/	National government
10	Brazil	https://www.semanticscholar.org/paper/ANA%3A-A-Brazilian-Chatbot-Assistant-about-COVID-19-Ferreira-Marcolino/6f254b3958a9080782a3c212b20b96f98909a8a1	Academics
11	Cambodia	http://geeksincambodia.com/new-facebook-messenger-chatbot-informs-cambodians-about-covid-19/	Private organisation(s) or individual(s)
12	Cameroon	https://www.digitalbusiness.africa/cameroun-digital-mama-le-chatbot-de-la-crtv-donnant-linfo-en-temps-reel-sur-levolution-du-covid-19-dans-le-pays/	TV channel/radio station

13	Canada	https://www2.gov.bc.ca/gov/content/about-gov-bc-ca/about-the-digital-assistant	National government
14	Chile	https://blog.investchile.gob.cl/chilean-company-develops-a-digital-solution-for-detecting-coronavirus	Private organisation(s) or individual(s)
15	Colombia	https://dotgo.com/bot_details/gobierno-de%20colombia%20covid-19/whatsapp?id=539	National government
16	Congo	https://actualite.cd/2020/10/12/le-chatbot-du-ministere-de-la-sante-pour-lutter-contre-la-desinformation-liee-covid-19	National government
17	Costa Rica	https://planetachatbot.com/asistente-virtual-sector-salud-costa-rica/	Private organisation(s) or individual(s)
18	Croatia	https://www.croatiaweek.com/croatias-infobip-wins-global-award-for-chatbot-solution/	Private organisation(s) or individual(s)
19	Czech Republic	https://services.global.ntt/zh-cn/newsroom/ntt-ltd-in-czech-republic-launches-koronabot-to-ease-workload	Private organisation(s) or individual(s)
20	Denmark	https://sifted.eu/articles/coronafiles-chatbots-helplines/	Private organisation(s) or individual(s)
21	Ecuador	https://1millionbot.com/chatbot-coronavirus-ecuador/	National government
22	Egypt	https://cairoscene.com/Buzz/Ask-Nameesa-Is-The-New-Egyptian-Chatbot-With-All-the-Answers-to-Your-Corona-Questions	National government
23	El Salvador	https://www.presidencia.gob.sv/gobierno-de-el-salvador-y-facebook-informan-a-la-comunidad-sobre-el-covid-19/	National government
24	Estonia	https://investinestonia.com/estonia-created-suve-an-automated-chatbot-to-provide-trustworthy-information-during-the-covid-19-situation/	National government
25	Ethiopia	https://www.thereporterethiopia.com/9564/	National government

26	Finland	https://koronabotti.hus.fi/en	Private organisation(s) or individual(s)
27	France	https://info.covidbot.fr/	National government
28	Georgia	https://eu4georgia.eu/c-bot-chatbot-with-ai-keeps-georgian-business-informed-during-pandemic/	National government
29	Germany	https://www.theee.ai/2020/04/09/1822-german-red-cross-launches-whatsapp-chatbot-to-answer-covid-19-questions/	National government
30	Ghana	https://www.undp.org/ghana/blog/crisis-mother-invention-mitigating-covid-19-through-innovation	National government
31	Guatemala	https://radiotgw.gob.gt/alma-el-chatbot-guatemalteco-para-resolver-dudas-sobre-el-covid-19/	Private organisation(s) or individual(s)
32	Hong Kong	https://www.coronavirus.gov.hk/eng/index.html	National government
33	Hungary	https://www.sap.com/hungary/products/technology-platform/use-cases/symptom-checker-chatbot.html	Private organisation(s) or individual(s)
34	India	https://tcrn.ch/2UplWab	National government
35	Indonesia	https://setkab.go.id/en/ministry-of-health-provides-whatsapp-chatbot-service-for-vaccination-registration/	National government
36	Iraq	https://www.telecompaper.com/news/unicef-reaches-14-mln-in-iraq-via-sms-with-critical-covid-19-info--1340777	UNICEF (U-report)
37	Ireland	https://www.hse.ie/eng/gdpr/data-protection-covid-19/dpin-hse-live-chat-bot.html	National government
38	Israel	https://www.bbc.co.uk/news/world-middle-east-55794283	National government
39	Italy	https://pharmaphorum.com/news/italian-startup-launches-coronavirus-diagnostic-chatbot	Regional government

40	Jamaica	https://www.unicef.org/innovation/stories/U-Report JamaicaCovid19Response	UNICEF (U-report)
41	Japan	https://www.reuters.com/article/us-health-coronavirus-japan-vaccine-idUSKBN2AC0BB	National government
42	Jordan	https://portal.jordan.gov.jo/wps/portal/Home/Chatbot	National government
43	Kazakhstan	https://www.thestar.com.my/tech/tech-news/2021/02/22/kazakhstan-enlists-chatbot-to-tackle-covid-19-vaccine-myths	Red Cross and IFRC
44	Kenya	https://dotgo.com/bot_details/ministry-of%20health%20kenya/whatsapp?id=450	National government
45	Kuwait	https://www.zawya.com/en/press-release/gilead-sciences-chatbot-to-support-healthcare-providers-in-kuwait-with-scientific-updates-on-covid-19-qu44t7e3	Private organisation(s) or individual(s)
46	Latvia	https://tilde.com/products-and-services/ai-powered-chatbots/case-studies/ai-powered-public-governance-latvia	National government
47	Lebanon	https://www.moph.gov.lb/en/view/35969/minister-hasan-launches-moph-chatbot-on-whatsapp-	National government
48	Liberia	https://www.facebook.com/unicef/photos/a.10150563250124002/10159856964614002	UNICEF (U-report)
49	Libya	https://www.wfp.org/stories/chatbot-named-mila-answering-call-people-libya	Private organisation(s) or individual(s)
50	Lithuania	https://oecd-opsi.org/covid-response/vilte-chatbot/	National government
51	Madagascar	https://dotgo.com/bot_details/mada-covid-19/whatsapp?id=1083&cid=790	National government
52	Malawi	https://itweb.africa/content/mYZRXv9aLrKvOgA8	National government
53	Malaysia	https://www.thestar.com.my/tech/tech-news/2020/07/14/health-ministry-to-launch-whatsapp-chatbot-to-answer-questions-on-covid-19	National government

54	Malta	https://lovinmalta.com/lifestyle/technology/maltese-doctors-set-up-facebook-chatbot-dedicated-to-answering-all-your-coronavirus-queries/	Private organisation(s) or individual(s)
55	Mexico	https://saluddigital.com/en/comunidades-conectadas/gobierno-de-mexico-lanza-chatbot-via-whatsapp-para-brindar-informacion-sobre-covid-19/	National government
56	Moldova	https://www.unicef.org/moldova/en/press-releases/cyber-hygiene-very-important-just-proper-handwashing	UNICEF (U-report)
57	Mongolia	https://www.unicef.org/media/81356/file/Mongolia-COVID19-SitRep-5-June-2020.pdf	National government
58	Morocco	https://www.boell.de/en/2020/04/07/digitalisierung-und-corona-marokko-zwischen-fuersorge-und-kontrolle	Private organisation(s) or individual(s)
59	Mozambique	https://www.itweb.co.za/content/JN1gPvOYBd5MjL6m ; https://globalvoices.org/2021/01/19/in-mozambique-a-tug-of-war-between-public-health-and-digital-rights-during-the-pandemic/	National government
60	Myanmar	https://www.pressreader.com/myanmar/the-myanmar-times/20200506/281629602437530	Private organisation(s) or individual(s)
61	Nepal	https://www.nepalitimes.com/latest/viber-and-unicef-nepal-join-against-coronavirus	UNICEF (U-report)
62	New Zealand	https://clearpoint.digital/consulting/innovation/whatsapp-chatbot-govt/	National government
63	Nicaragua	https://nextbillion.net/calendar/chat-for-impact-in-the-time-of-covid/	News platform
64	Niger	https://www.unicef.org/niger/press-releases/niger-launches-chatbot-whatsapp-answer-covid-19-queries	National government
65	Nigeria	https://www.unicef.org/nigeria/press-releases/ncdc-and-unicef-launch-chatbot-combat-covid-19-misinformation-nigeria	National government
66	North Macedonia	https://eurohealthobservatory.who.int/news-room/articles/item/public-health-in-europe-in-times-of-covi	UNICEF and WHO (HealthBuddy+)

		d-19-country-snapshot-on-the-role-of-public-health-agencies-and-services-in-north-macedonia	
67	Norway	https://www.nav.no/kontaktoss/en	National government
68	Oman	https://action.bot/case-studies/chatbot-for-the-public-sector/	National government
69	Pakistan	https://www.medianama.com/2020/04/223-pakistan-whatsapp-chatbot-coronavirus/	National government
70	Peru	https://rpp.pe/peru/actualidad/coronavirus-crean-primer-chatbot-que-responde-consultas-sobre-la-covid-19-las-24-horas-del-dia-noticia-1268863	National government
71	Philippines	https://www.bworldonline.com/health/2022/09/07/472821/pinaslakas-ai-chatbot-boost-vax-efforts/	National government
72	Poland	https://pacjent.gov.pl/aktualnosc/wirtualny-asystent-odpowie-na-pytania	National government
73	Qatar	https://www.gco.gov.qa/en/2020/04/government-communications-office-launches-coronavirus-information-service-on-whatsapp/	National government
74	Romania	https://gov.ro/ro/stiri/conferinta-de-presa-sustinuta-de-valeriu-gheorghita-pre-edintele-comitetului-national-de-coordonare-a-activitatilor-privind-vaccinarea-impotriva-sars-cov-2-cncav-i-andrei-baciu-secretar-de-stat-in-ministerul-sanatatii-vicepre-edinte-al-cncav1627387584	UNICEF and WHO (HealthBuddy+)
75	Russia	https://minzdrav.gov.ru/news/2020/04/09/13686-minzdrav-rossii-zapustil-chat-bot-dlya-informirovaniya-naseleniya-o-covid-19	National government
76	Rwanda	https://international-partnerships.ec.europa.eu/policies/programming/projects/mbaza-chatbot-giving-access-covid-19-information-throughout-rwanda_en	National government
77	Saudi Arabia	https://dotgo.com/bot_details/seha/whatsapp?id=1518&cid=1193	Private organisation(s) or individual(s)
78	Senegal	https://www.adie.sn/actualites/lutte-contre-la-covid19-l%E2%80%99adie-arme-le-minist%C3%A8re-de-la-sant%C3%A9-avec-une-plateforme-un	National government

79	Serbia	https://www.weaverbot.ai/blog/covid-19-chatbot	National government
80	Singapore	https://www.developer.tech.gov.sg/products/categories/digital-solutions-to-address-covid-19/covid-19-chatbots/overview.html	National government
81	Slovakia	https://www.slovensko.sk/sk/oznamy/detail/_covid-19-potrebne-informacie	National government
82	Slovenia	https://the-slovenia.com/health-fitness/coronavirus-in-slovenia-vid-chatbot/	Private organisation(s) or individual(s)
83	Somalia	https://acclabsomalia.medium.com/new-somali-whatsapp-chatbot-helps-in-the-fight-against-covid-19-47b40a9f1d79	Private organisation(s) or individual(s)
84	South Africa	https://www.itweb.co.za/content/JN1gPvOYBd5MjL6m	National government
85	South Korea	https://www.aitimes.kr/news/articleView.html?idxno=15403	Private organisation(s) or individual(s)
86	Spain	https://www.lamoncloa.gob.es/serviciosdeprensa/notasprensa/asuntos-economicos/Paginas/2020/080420-consulta.aspx	National government
87	Sri Lanka	https://airtable.com/shrGXLJECotnZa1Ou/tblwPhDJfiisTMNg6/viwG94eM2oA9pZuib/recTZoPONQb78Kzeh?blocks=bipVDslkfppjON6Dh	UNDP
88	Sweden	https://www.boost.ai/case-studies/vgr-conversational-ai-covid-case-study	Regional government
89	Switzerland	https://www.rafael-postcovid.ch/	Private organisation(s) or individual(s)
90	Taiwan	https://focustaiwan.tw/sci-tech/202005220015	National government
91	Thailand	https://www.bangkokpost.com/thailand/general/2268399/chatbot-helps-assess-covid-19-symptoms	National government
92	Togo	https://www.togofirst.com/en/itc/1205-5518-covid-19-government-s-chatbot-dokita-is-now-operational-on-whatsapp	National government

93	Tunisia	https://www.lemonde.fr/afrique/article/2020/06/02/en-tunisie-un-chatbot-pour-contrer-le-coronavirus_6041546_3212.html	Private organisation(s) or individual(s)
94	UAE	https://u.ae/en/information-and-services/justice-safety-and-the-law/handling-the-covid-19-outbreak/smart-solutions-to-fight-covid-19/virtual-doctor-for-covid-19	National government
95	Uganda	https://www.dotgo.com/bot_details/covid-19%20uganda/whatsapp?id=1078&cid=785	National government
96	UK	https://www.gov.uk/government/news/government-launches-coronavirus-information-service-on-whatsapp	National government
97	Ukraine	https://basicgroup.ua/ministry-of-health-restarting-the-chat-bot-on-koronavirus/	National government
98	Uruguay	https://saluddigital.com/en/comunidades-conectadas/chatbot-del-gobierno-uruguayo-para-canalizar-posibles-casos-de-covid-19-recibe-importante-actualizacion/	National government
99	USA	https://www.naccho.org/blog/articles/three-u-s-health-departments-awarded-in-the-vira-chatbot-competition-health-departments-will-use-custom-ai-chatbots-to-address-covid-19-misinformation, https://www.microsoft.com/en-us/industry/microsoft-in-business/customer-experience/2020/06/24/how-the-washington-department-of-health-used-a-self-service-interactive-chatbot-built-on-ai-to-keep-the-public-educated/	Multiple state governments
100	Venezuela	https://www.codeforvenezuela.org/projects	Private organisation(s) or individual(s)
101	Vietnam	https://www.sggpnews.org.vn/chatbot-updating-citizens-on-covid-19-information-daily-post82635.html	National government
102	Wales	https://www.computerweekly.com/news/252481083/Coronavirus-Welsh-National-Health-Service-preps-Watson-powered-chatbot	National government
		https://racecouncilcymru.org.uk/government-launch	National

		es-coronavirus-information-service-on-whatsapp	government
103	Zambia	https://www.engati.com/blog/chatbot-case-study-government	National government
104	Zimbabwe	https://dotgo.com/bot_details/zimbabwe-govt/whatsapp?id=635	National government

Supplementary Table 1. Identified COVID-19 chatbots. This table outlines the set of COVID-19 chatbots that we could identify by conducting a Google search using the following search terms: ‘country name + COVID-19 chatbot’, ‘country name + coronavirus chatbot’ and ‘country name + COVID-19 chatbot + ministry of health’ in English and the national language of the country. This table is not intended to be an exhaustive review of all chatbots produced during the pandemic, but indicative of the scale of the use of chatbots. A chatbot was considered to have a government provider/developer if it was developed by a government or government-affiliated body, hosted on a government website or formally endorsed or named as a government chatbot. Chatbots that did not meet these conditions but repeated government guidelines or were informed by government guidance were not considered government chatbots. The UNDP, UNICEF and the WHO also developed chatbots specifically for COVID-19 which were rolled out in many countries. As these chatbots fall out of use, some of the URLs listed here may become defunct.

Description of chatbot functionality and message details	Screenshot of chatbot conversation
**Functionality 1 - Personalised eligibility**<br><br>Users first received a personalised introductory message that addressed them by name, stated the date of their last dose of the vaccine, which number dose it was and confirmed that they were now eligible to receive the next dose.<br><br>This message also introduced the service and stated its affiliation with the Ministry of Health.<br><br>It contained a motivational statement that aimed to invoke loss aversion, which translates to English as 'Over time, your defences against the virus decrease. Reinforce your vaccine to avoid losing your protection against the virus'. This motivational statement was also included in the one-way message trial arm.<br><br>Out of view in this screenshot was an additional statement which informed the user about where they could get more information. This statement did not contain a URL, as focus groups suggested a link would lower trust in the service. We also informed users that if they did not reply to the message, they would not be messaged again.<br><br>Finally, users were given the option to click continue to opt-in to using the service. If they did so, they were asked to confirm that the details provided in the introductory message were correct.	Hola, Dan Brown 👋<br><br>Este es un nuevo servicio del Ministerio de Salud para recibir información sobre tu próxima vacuna gratuita contra el Covid-19 🦠<br><br>Según el registro del Ministerio, te diste la 1ra dosis de la vacuna el 01/02/2022 y ya te toca tu 2da dosis.<br><br>Con el paso del tiempo, tus defensas contra el virus disminuyen. Refuerza tu vacuna y no pierdas tu protección contra el virus!<br><br>En este chat te podemos ayudar a conocer cuándo y dónde recibir tu próxima vacuna. **¿Querés continuar?**

Description of chatbot functionality and message details	Screenshot of chatbot conversation
**Functionality 2 - Health centre locator (Step 1)**<br><br>Next, the chatbot allowed users to find the health centres closest to their location, either by sharing their current location using WhatsApp's 'Share Location' function or by entering their 4-digit postcode.<br><br>We gave users the option to share their postcode in addition to the Share Location option because: 1) the latter may be perceived as private, 2) some users may wish to see health centre options for somewhere other than their current location, e.g., see centres near their work whilst accessing the chatbot from home, and, 3) we did not want to alienate users who did not know how to share their current location.<br><br>We found that of those who chose to share their location with the chatbot, 60% used the postcode option and 40% used WhatsApp's Share Location function.	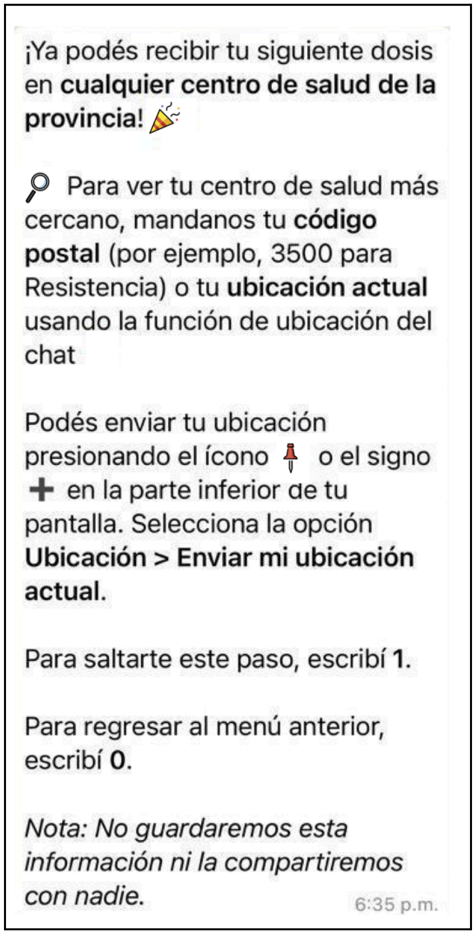

Description of chatbot functionality and message details	Screenshot of chatbot conversation
**Functionality 2 - Health centre locator (Step 1, Resistencia and Sáenz Peña)**<br><br>In two densely populated cities, Resistencia and Presidencia Roque Sáenz Peña, there were many health centres in each postcode.<br><br>To avoid showing users a very long list of health centres, we presented any user who entered a postcode associated with either city an intermediate step: a map of their city, divided into numbered sections. From here, they could select the division for which they wanted to see available health centres.	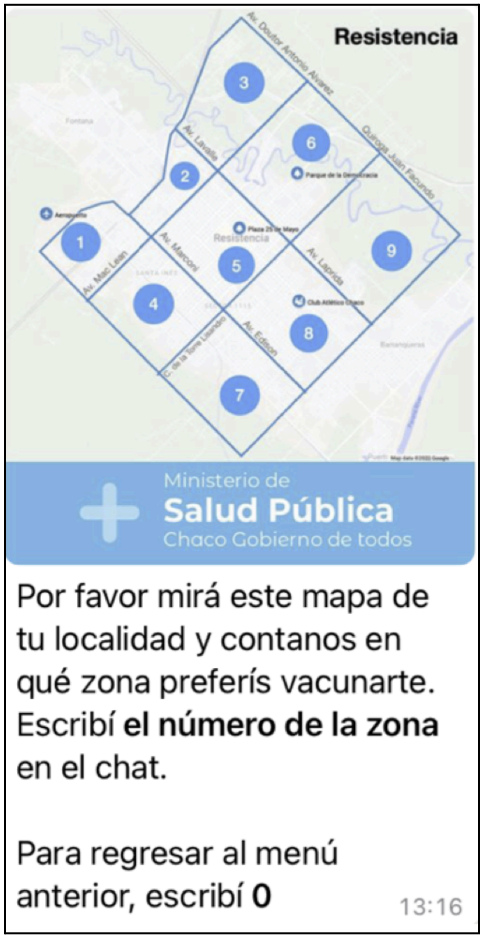

Description of chatbot functionality and message details	Screenshot of chatbot conversation
**Functionality 2 - Health centre locator (Step 2)**<br><br>Using the location information, the chatbot then retrieved details of the closest health centres, including their addresses and opening hours.<br><br>Those who used WhatsApp’s Share Location function were shown their three closest centres (determined using the coordinates of their current location and those of the health centres).<br><br>Those who shared their postcode received details of all health centres in the postcode, up to a maximum of 5 (the two postcodes with more than 5 health centres were dealt with as described in the previous step).<br><br>Once users had been sent a numbered list of health centres, they were asked to select the centre they would like to visit, which started the planning prompt functionality.	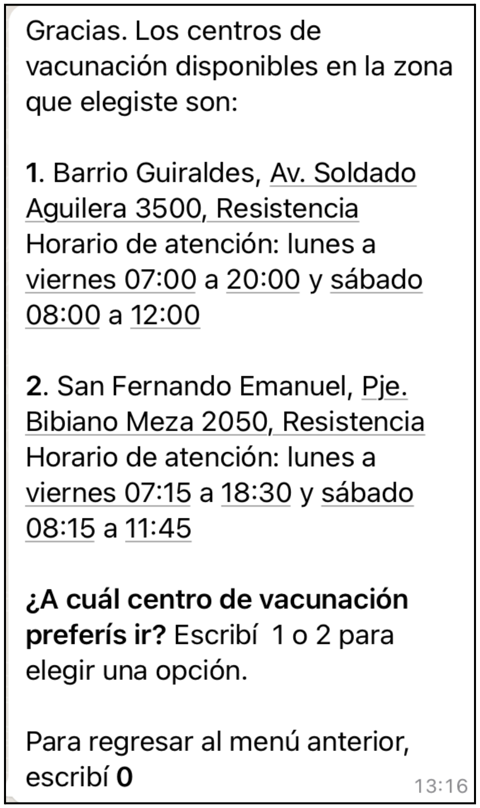<br>Gracias. Los centros de vacunación disponibles en la zona que elegiste son:<br><br>**1.** Barrio Guiraldes, Av. Soldado Aguilera 3500, Resistencia<br>Horario de atención: lunes a viernes 07:00 a 20:00 y sábado 08:00 a 12:00<br><br>**2.** San Fernando Emanuel, Pje. Bibiano Meza 2050, Resistencia<br>Horario de atención: lunes a viernes 07:15 a 18:30 y sábado 08:15 a 11:45<br><br>**¿A cuál centro de vacunación preferís ir?** Escribí 1 o 2 para elegir una opción.<br><br>Para regresar al menú anterior, escribí **0**<br>13:16

Description of chatbot functionality and message details	Screenshot of chatbot conversation
**Functionality 3 - Planning prompt (Step 1)**<br><br>After indicating their chosen health centres, users were prompted to make a personal plan for when they will get the vaccine. In the first step, they entered their chosen date.	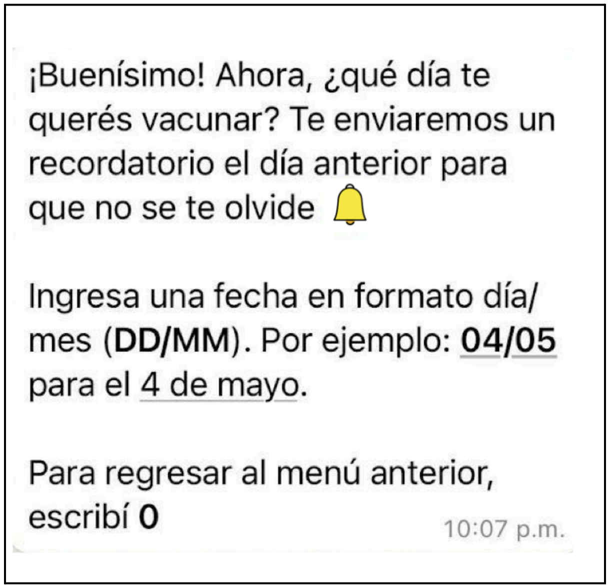

Description of chatbot functionality and message details	Screenshot of chatbot conversation
**Functionality 3 - Planning prompt (Step 2)**<br><br>In the second step, they entered the time of day they would get the vaccine.<br><br>If users answered both of the planning prompts, a reminder was programmed and the user received a confirmation that they would be reminded the day before.	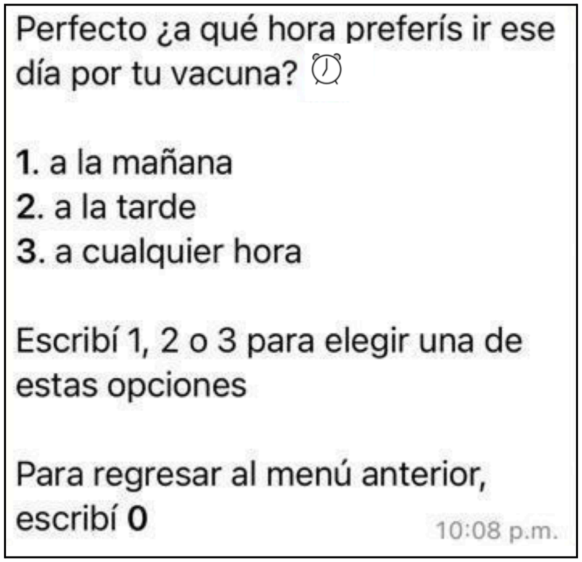

<table>
<tr><th>Description of chatbot functionality and message details</th><th>Screenshot of chatbot conversation</th></tr>
<tr><td>Functionalities 4 and 5 - Reminder and directions

At 6pm on the evening before their selected day of vaccination, users who had reached the end of the chatbot flow received an automated and personalised reminder message. It included the name of the selected health centre, its address, opening hours, and a link to a GoogleMaps shortcut providing directions from their current location to the centre (if they have permitted location sharing in GoogleMaps).

In limited circumstances, users did not receive a reminder message. Specifically, if individuals responded to the original chatbot after 6pm and chose the next day as their vaccination date, they would not receive a reminder message because the script for reminder messages that day had already been executed. As such, 400 out of the 3,705 chatbot recipients who completed all steps in the chatbot flow did not receive a reminder message. Given that these individuals intended to get the vaccination the next day, we are not concerned by the lack of reminder, but this does mean they did not receive directions to their chosen health centre.</td><td>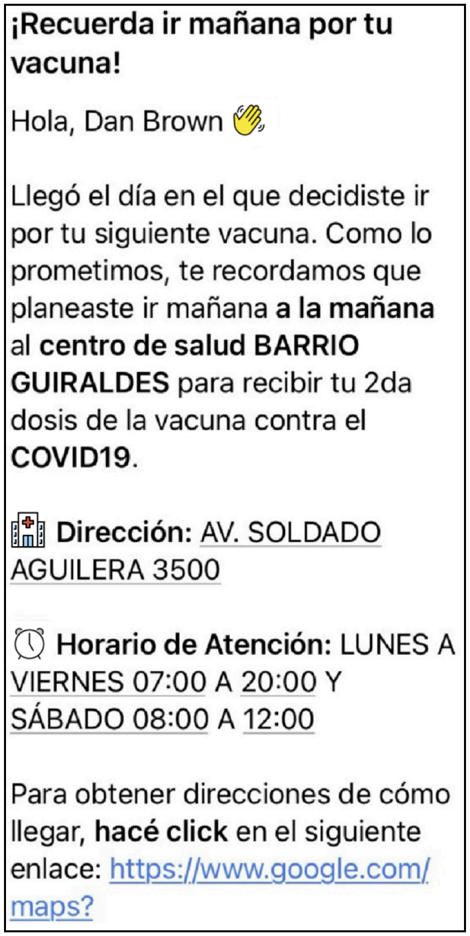
</td></tr>
</table>

Supplementary Table 2. Intervention design. This table describes the most direct path through the chatbot flow (referred to as the ‘happy path’). The left column explains how each of the functionalities work, and the right column provides a screenshot of the corresponding message. The chatbot was sent from Chaco Ministry of Health’s official phone line, with its logo displayed in the top left corner, and a WhatsApp verified green tick in the top right corner. The images shown were modified from the originals in order to replace copyrighted material.

Covariate	Test (1)	Mean for control (2)	Mean for one-way message (3)	Mean for chatbot (4)	p-value: control vs. one-way message (5)	p-value: control vs. chatbot (6)	p-value: one-way message vs. chatbot (7)
Sex	Two-proportions test	48.3% male	48.5% male	48.6% male	0.292	0.144	0.688
Age	Chi-square	18-29: 28.2% 30-49: 47.5% 50+: 24.2%	18-29: 28.0% 30-49: 47.6% 50+: 24.4%	18-29: 27.9% 30-49: 47.7% 50+: 24.4%	0.551	0.221	0.768
Number of doses before entering trial	Chi-square	1: 12.1% 2: 46.9% 3: 40.3% 4: 0.6%	1: 12.1% 2: 46.9% 3: 40.3% 4: 0.6%	1: 12.1% 2: 46.9% 3: 40.3% 4: 0.6%	1	1	1
Days between last dose before randomisation and 8 Sep 2022	Two-sample t-test	310.6	311	311.2	0.37	0.198	0.695
Days from 8 Sep 2022 that message received	Two-sample t-test	7.36	7.35	7.36	0.829	0.928	0.76

Supplementary Table 3. Balance tests. This table reports the results from balance tests for the sample of 249,705 participants used in the primary analysis. In column (1) we report the test used for the relevant covariate; all tests are two-sided. Columns (2), (3) and (4) report the mean for the control group, one-way message group and chatbot group respectively. Columns (5), (6) and (7) report p-values from pairwise comparisons between: control and one-way message, control and chatbot, and one-way and chatbot groups respectively. The date that the message was received was imputed for the control group as the date that they were enrolled into the trial, as outlined in the implementation procedure described in the materials and methods.

	Vaccinations within 4 weeks				
	Male	Female	Age-18-29	Age-30-49	Age-50+
	(1)	(2)	(3)	(4)	(5)
One-way message	0.732***	0.513***	0.418***	0.557***	0.826***
	(<0.001)	(<0.001)	(<0.001)	(<0.001)	(<0.001)
	[0.568, 0.896]	[0.383, 0.643]	[0.213, 0.623]	[0.410, 0.704]	[0.630, 1.022]
Chatbot	1.452***	0.993***	0.720***	1.120***	1.606***
	(<0.001)	(<0.001)	(<0.001)	(<0.001)	(<0.001)
	[1.302, 1.602]	[0.872, 1.114]	[0.526, 0.915]	[0.985, 1.256]	[1.426, 1.786]
Observations	121,064	128,641	69,990	118,913	60,802
Control-group mean	0.53%	0.86%	0.66%	0.71%	0.72%
p-value: one-way message vs. chatbot	<0.001	<0.001	0.001	<0.001	<0.001
Estimated treatment effect one-way message (ppts)	0.57pp	0.57pp	0.34pp	0.52pp	0.91pp
Estimated treatment effect chatbot (ppts)	1.70pp	1.42pp	0.69pp	1.44pp	2.76pp

Supplementary Table 4. Subgroup analysis by demographic characteristics. Columns (1) and (2) present regression results for men and women respectively, and columns (3), (4) and (5) present regression results by age group as indicated. In all cases, we used a multivariate logistic regression and we controlled for gender (other than columns (1) and (2)), age (other than columns (3)-(5)), the number of COVID-19 doses already received, time since previous dose and the date of message delivery. The dependent variable was a binary indicator for whether the individual received their next dose of the COVID-19 vaccine within a four week period. The estimated treatment effects in the final two rows are the effect at the control group mean. We use two-sided z-tests, and report p-values in parentheses. We do not adjust p-values for multiple comparisons in this exploratory sub-group analysis. *** indicates statistical significance at the 0.1% level. 95% confidence intervals are shown in square brackets.

	Gender and age only	No covariates	OLS	Probit	Firth's	King and Zeng's	2 weeks	6 weeks
	(1)	(2)	(3)	(4)	(5)	(6)	(7)	(8)
One-way message	0.603***	0.603***	0.006***	0.221***	0.598***	0.598***	0.741***	0.533***
	(<0.001) [0.501, 0.705]	(<0.001) [0.502, 0.705]	(<0.001) [0.005, 0.007]	(<0.001) [0.183, 0.258]	(<0.001) [0.497, 0.700]	(<0.001) [0.496, 0.700]	(<0.001) [0.614, 0.867]	(<0.001) [0.444, 0.622]
Chatbot	1.189***	1.189***	0.016***	0.453***	1.186***	1.186***	1.447***	1.036***
	(<0.001) [1.096, 1.283]	(<0.001) [1.096, 1.283]	(<0.001) [0.014, 0.017]	(<0.001) [0.418, 0.488]	(<0.001) [1.093, 1.280]	(<0.001) [1.092, 1.279]	(<0.001) [1.331, 1.562]	(<0.001) [0.954, 1.118]
Observations	249,705	249,705	249,705	249,705	249,705	249,705	249,705	249,705
Control-group mean	0.70%	0.70%	0.70%	0.70%	0.70%	0.70%	0.43%	0.94%
Adjusted p-value: one-way message vs. chatbot	<0.001	<0.001	<0.001	<0.001	<0.001	<0.001	<0.001	<0.001
Estimated treatment effect one-way message (ppts)	0.57pp	0.57pp	0.57pp	0.57pp	0.57pp	0.57pp	0.47pp	0.65pp
Estimated treatment effect chatbot (ppts)	1.56pp	1.56pp	1.56pp	1.55pp	1.55pp	1.55pp	1.37pp	1.67pp

Supplementary Table 5. Robustness checks. In all columns we re-ran the main regression with a change in either the specification or the outcome measure to check the robustness of the main results. Column (1) drops all covariates other than age and gender, and column (2) drops all covariates. In column (3), we ran a linear probability model (and reported robust standard errors), and in column (4) a Probit model. To correct for rare events in binary data, in columns (5) and (6) we use King and Zeng's correction for logistic regression and Firth's penalised likelihood approach, respectively (*1, 2*). In columns (7) and (8), the dependent variable was a binary variable indicating whether the individual got their next dose of the COVID-19 vaccination within two or six weeks respectively. The estimated treatment effects in the final two rows are the effect at the control group mean. We use two-sided z-tests, and report adjusted p-values which use the Benjamini-Hochberg procedure to correct for all 3 between-arm comparisons in parentheses. *** indicates statistical significance at the 0.1% level. 95% confidence intervals are shown in square brackets.

	Vaccinations within 4 weeks		
	1 Dose	2 Doses	3 Doses
	(1)	(2)	(3)
One-way message	0.431*	0.498***	0.682***
	(0.020)	(<0.001)	(<0.001)
	[0.069, 0.793]	[0.332, 0.664]	[0.542, 0.821]
Chatbot	0.501**	1.005***	1.365***
	(0.006)	(<0.001)	(<0.001)
	[0.144, 0.859]	[0.852, 1.158]	[1.237, 1.493]
Observations	30,208	117,158	100,723
Control-group mean	0.50%	0.58%	0.89%
p-value: one-way message vs. chatbot	0.671	<0.001	<0.001
Estimated treatment effect one-way message (ppts)	0.27pp	0.37pp	0.86pp
Estimated treatment effect chatbot (ppts)	0.32pp	0.99pp	2.52pp

Supplementary Table 6. Subgroup analysis by number of COVID-19 vaccine doses prior to trial. In this table, we re-ran the main regression for three subgroups: those who had previously had 1, 2 or 3 doses of the COVID-19 vaccine before the launch of the trial. Only 1,616 individuals had received 4 doses of the vaccine before the launch of the trial, and so we did not run a separate regression for that subgroup. In all cases, we used a logistic regression and we controlled for gender, age, time since previous dose and the date of message delivery. The dependent variable was a binary indicator for whether the individual received their next dose of the COVID-19 vaccine within a four week period. The estimated treatment effects in the final two rows are the effect at the control group mean. We use two-sided z-tests and report p-values in parentheses. We do not adjust p-values for multiple comparisons in this exploratory sub-group analysis. ***, ** and * indicate statistical significance at the 0.1%, 1% and 5% levels respectively. 95% confidence intervals are shown in square brackets.

Supplemental References

1. King, G. & Zeng, L. Logistic regression in rare events data. Pol. Anal. 9, 2, 137-163 (2001)
2. Suhas, S., Manjunatha, N., Kumar, C. N., Benegal, V., Rao, G.N., Varghese, M. & Gururaj, G. Firth's penalized logistic regression: A superior approach for analysis of data from India's National Mental Health Survey. Indian Journal of Psychiatry 65, 12, 1208-1213 (2023).
