# Supplementary material for: A behaviourally informed chatbot increases vaccination rates in Argentina more than a one-way reminder
Source: Nat Hum Behav. 2024 Oct 18;8(12):2314–21. doi: 10.1038/s41562-024-01985-7 (PMC11659163; doi:10.1038/s41562-024-01985-7)
Supplement: Supplementary file 2 — Reporting Summary [file 41562_2024_1985_MOESM2_ESM.pdf]

nature portfolio

Corresponding author(s): Dan Brown

Last updated by author(s): 30/06/24

Reporting Summary

Nature Portfolio wishes to improve the reproducibility of the work that we publish. This form provides structure for consistency and transparency in reporting. For further information on Nature Portfolio policies, see our Editorial Policies and the Editorial Policy Checklist.

Statistics

For all statistical analyses, confirm that the following items are present in the figure legend, table legend, main text, or Methods section.

n/a	Confirmed	
☐	☒	The exact sample size (*n*) for each experimental group/condition, given as a discrete number and unit of measurement
☐	☒	A statement on whether measurements were taken from distinct samples or whether the same sample was measured repeatedly
☐	☒	The statistical test(s) used AND whether they are one- or two-sided<br>*Only common tests should be described solely by name; describe more complex techniques in the Methods section.*
☐	☒	A description of all covariates tested
☐	☒	A description of any assumptions or corrections, such as tests of normality and adjustment for multiple comparisons
☐	☒	A full description of the statistical parameters including central tendency (e.g. means) or other basic estimates (e.g. regression coefficient) AND variation (e.g. standard deviation) or associated estimates of uncertainty (e.g. confidence intervals)
☐	☒	For null hypothesis testing, the test statistic (e.g. *F*, *t*, *r*) with confidence intervals, effect sizes, degrees of freedom and *P* value noted<br>*Give P values as exact values whenever suitable.*
☒	☐	For Bayesian analysis, information on the choice of priors and Markov chain Monte Carlo settings
☒	☐	For hierarchical and complex designs, identification of the appropriate level for tests and full reporting of outcomes
☒	☐	Estimates of effect sizes (e.g. Cohen's *d*, Pearson's *r*), indicating how they were calculated

Our web collection on statistics for biologists contains articles on many of the points above.

Software and code

Policy information about availability of computer code

Data collection	No software was used for data collection. The intervention was implemented using WhatsApp.
Data analysis	Data analysis was conducted using R version 4.4.0.

For manuscripts utilizing custom algorithms or software that are central to the research but not yet described in published literature, software must be made available to editors and reviewers. We strongly encourage code deposition in a community repository (e.g. GitHub). See the Nature Portfolio guidelines for submitting code & software for further information.

Data

Policy information about availability of data

All manuscripts must include a data availability statement. This statement should provide the following information, where applicable:

- Accession codes, unique identifiers, or web links for publicly available datasets
- A description of any restrictions on data availability
- For clinical datasets or third party data, please ensure that the statement adheres to our policy

We used data from the NOMIVAC vaccinations database (Registro Federal de Vacunación Nominalizado). The data used in the analysis are publicly available in the Dryad data repository at the following link: https://datadryad.org/stash/share/HPMMIkG8ltPniOJJChLa6ci81SaDKXK24I68g8njXCk. No accession code is required. The data are anonymity-preserving.

Research involving human participants, their data, or biological material

Policy information about studies with human participants or human data. See also policy information about sex, gender (identity/presentation), and sexual orientation and race, ethnicity and racism.

Reporting on sex and gender	Data on sex was used in the analysis. This data was collected from an administrative dataset. Of the 249,705 individuals in the sample, 48.5% were male and 51.5% female. Analysis was conducted by sex, as reported in Table S4 in the Supplementary Materials. The effect of both the one-way message and the chatbot treatment was statistically significant for both men and women: a 0.57ppt and 1.70ppt effect for men respectively, and a 0.57ppt and 1.42ppt effect for women respectively.
Reporting on race, ethnicity, or other socially relevant groupings	Data on race, ethnicity or other socially relevant groupings were not used in the study.
Population characteristics	In addition to data on sex, data on age and previous COVID-19 vaccinations was collected through administrative databases and used both as covariates for the regression analysis and for the sub-group analysis. Analysis by age and by previous number of COVID-19 vaccine doses is reported in Tables S4 and S6 in the Supplementary Materials respectively.
Recruitment	We recruited adults in Chaco province, Argentina, who were eligible to receive their next dose of the COVID-19 vaccine. Further details on eligibility criteria at the time of the study are outlined in the Supplementary Materials. To do this, we first constructed a database of potential study participants using phone numbers from three administrative data sources provided by the Ministry of Health (Pasaporte Chaco, SUMAR, and a 0800 helpline). Further details on each data source is provided in the Methods section. From this database, we included any individual in the eligible sample who: i) Had received the first COVID-19 vaccine dose, ii) Was eligible to receive their next dose; i.e., the 2nd, 3rd, 4th, or 5th dose of the COVID-19 vaccine, iii) Was 18 years of age or older, iv) Had a mobile number registered with WhatsApp, v) Had a mobile number that was unique to one individual within the study database, and vi) Had only one mobile phone registered within the study database. This provided us with a final sample of 249,705 participants. Given that we explicitly sampled individuals who had already received one dose of the COVID-19 vaccine at the time that the project launched, the results should not be interpreted as representative of the effect for the whole Chaco population (the effect of the chatbot could have been larger or smaller for people who had not yet received any doses).
Ethics oversight	The study was assessed by an independent research ethics committee at Favaloro University Hospital (Comité de Bioética, Fundación Favaloro Hospital Universitario: CBE Acta Nº 121, 29/06/2022) and approved prior to implementation.

Note that full information on the approval of the study protocol must also be provided in the manuscript.

Field-specific reporting

Please select the one below that is the best fit for your research. If you are not sure, read the appropriate sections before making your selection.

☐ Life sciences ☒ Behavioural & social sciences ☐ Ecological, evolutionary & environmental sciences

For a reference copy of the document with all sections, see nature.com/documents/nr-reporting-summary-flat.pdf

Behavioural & social sciences study design

All studies must disclose on these points even when the disclosure is negative.

Study description	Randomised controlled trial. The data is quantitative.
Research sample	Our research sample comprised adults (individuals aged 18 years and older) in Chaco province, Argentina, who were eligible to receive their next dose of the COVID-19 vaccine, and who met the other inclusion criteria described in the recruitment section above. As stated above, we restricted the sample to only include individuals who had received at least one dose of the COVID-19 vaccine. Individuals who had not received any doses by this point in the pandemic were likely to be unwilling to get vaccinated and so we believe they would not benefit from a chatbot service. However, shortly before the launch of the trial, 83% of the population in Chaco had received at least one dose of the vaccine. Of the 249,705 individuals in the sample, 48.5% were male and 51.5% female, 28.0% were between 18-29 years old, 47% were between 30-49 and 24.3% were 50 or older. The sample is not representative of the whole Chaco adult population: it does not include any individuals who had not yet received any doses of the COVID-19 vaccine, and it skews younger than the overall Chaco adult population (for whom the relevant proportions are: 47.6% male and 52.4% female, with 29.3% aged 18-29, 39.5% aged 30-49 and 31.2% aged 50 or older).
Sampling strategy	We included all individuals who met our eligibility criteria (described in the recruitment section above), which gave us a final sample size of 249,705 participants. Our sampling strategy was to include all eligible individuals. We conducted statistical power calculations based on this anticipated sample size, and compared the resulting minimum detectable effect size (approximately a 0.14 percentage point effect) to effect sizes of a comparable intervention (text message reminders) in the existing academic literature (which ranged from 0.7ppts to 7.4ppts) to ensure that the trial was adequately powered before we began.
Data collection	Data on our outcome measure was taken from an existing administrative dataset of all COVID-19 vaccinations, called NOMIVAC, which was provided to us by the Ministry of Health. We did not do any primary data collection for the outcome measure. The researchers were not blind to the experimental conditions or the study hypotheses.

Timing	Data for the primary outcome measure was taken from September 9th 2022 to October 19th 2022. The data collection period was extended by a further two weeks to 2nd November 2022 to create the outcome measure for one of the robustness checks.
Data exclusions	No data were excluded from the final sample of 249,705 participants described in the recruitment section above.
Non-participation	Our outcome measure is based on a complete administrative dataset of all vaccinations, and so there is no attrition from the sample.
Randomization	Participants were allocated randomly to the three experimental groups using a random number generator. Randomisation was stratified by the number of COVID-19 vaccine doses already received by the individual (1, 2, 3 or 4).

Reporting for specific materials, systems and methods

We require information from authors about some types of materials, experimental systems and methods used in many studies. Here, indicate whether each material, system or method listed is relevant to your study. If you are not sure if a list item applies to your research, read the appropriate section before selecting a response.

Materials & experimental systems

n/a	Involved in the study
☒	☐ Antibodies
☒	☐ Eukaryotic cell lines
☒	☐ Palaeontology and archaeology
☒	☐ Animals and other organisms
☒	☐ Clinical data
☒	☐ Dual use research of concern
☒	☐ Plants

Methods

n/a	Involved in the study
☒	☐ ChIP-seq
☒	☐ Flow cytometry
☒	☐ MRI-based neuroimaging

Plants

Seed stocks	*Report on the source of all seed stocks or other plant material used. If applicable, state the seed stock centre and catalogue number. If plant specimens were collected from the field, describe the collection location, date and sampling procedures.*
Novel plant genotypes	*Describe the methods by which all novel plant genotypes were produced. This includes those generated by transgenic approaches, gene editing, chemical/radiation-based mutagenesis and hybridization. For transgenic lines, describe the transformation method, the number of independent lines analyzed and the generation upon which experiments were performed. For gene-edited lines, describe the editor used, the endogenous sequence targeted for editing, the targeting guide RNA sequence (if applicable) and how the editor was applied.*
Authentication	*Describe any authentication procedures for each seed stock used or novel genotype generated. Describe any experiments used to assess the effect of a mutation and, where applicable, how potential secondary effects (e.g. second site T-DNA insertions, mosiacism, off-target gene editing) were examined.*
